# Impact of Pharmacovigilance Sensitization on Knowledge and Attitude Amongst Medical and Paramedical Students in a Tertiary Care Teaching Hospital: A Questionnaire-Based Study

**DOI:** 10.7759/cureus.94002

**Published:** 2025-10-07

**Authors:** Vaishali Thakare, Mukta Jain, Masum Reza, Anant Patil, Deepak Langade

**Affiliations:** 1 Pharmacology, DY Patil University School of Medicine, Navi Mumbai, IND; 2 Pharmacology, Mahatma Gandhi Mission's (MGM) Medical College, Navi Mumbai, IND

**Keywords:** adr reporting systems, adverse drug reactions (adr), knowledge attitude practice (kap), medical and para-medical students, medical education, national pharmacovigilance week (npw), pvpi sensitization

## Abstract

Introduction

Adverse drug reactions (ADRs) are a major cause of morbidity, mortality, and healthcare burden worldwide. Underreporting of ADRs is a major barrier to drug safety, making early sensitization of healthcare students through pharmacovigilance programmes essential. Sensitization during undergraduate training of medical and para-medical students is therefore important to instill early awareness, bridge knowledge gaps, and encourage proactive participation in ADR reporting. The objective of this study was to evaluate the knowledge, attitude, and impact of a pharmacovigilance sensitization session among medical and paramedical students.

Methods

A pre-post interventional study using a validated knowledge-attitude questionnaire was conducted during the fourth National Pharmacovigilance Week (2024) at a tertiary care teaching hospital. Participants (medical, Ayurveda, and paramedical students) completed pre- and post-test surveys. Data were analyzed using descriptive statistics and chi-square tests. Data was analysed with the help of IBM SPSS Statistics for macOS, Version 29.0 (IBM Corp., Armonk, NY, USA).

Results

A total of 259 students participated (130 medical/ayurveda and 129 paramedical) in the study. Pre-test knowledge was significantly higher in medical students regarding ADR reporting (n=107, 82.5% vs. n=81, 62.8%; p=0.0007) and ADR forms (n=80, 62% vs. n=63, 48.8%; p=0.0536). Post intervention, both groups showed significant improvements, especially in recognizing pharmacovigilance terminology (n=71, 87.7% and n=57, 66.3%) and ADR reporting forms (n=49, 98% and n=52, 78.8%). Attitudes also improved, with medical students consistently demonstrating more positive responses.

Conclusion

Sensitization sessions during the National Pharmacovigilance Week significantly improved knowledge and attitudes toward pharmacovigilance and ADR reporting among future healthcare providers. Regular interventions are recommended to sustain awareness.

## Introduction

Adverse drug reactions (ADRs) remain a major, largely preventable cause of patient morbidity, mortality, and increased healthcare costs worldwide. The World Health Organization (WHO) defines an ADR as a harmful, unintended response to a drug administered at standard doses for prophylaxis, diagnosis, or therapy [1]. Despite the centrality of pharmacovigilance to medication safety, underreporting of ADRs persists across health systems. Among available methods, voluntary (spontaneous) reporting is the cornerstone of effective pharmacovigilance because it enables the detection of rare, unexpected, or serious reactions in real-world use [2].

Healthcare professionals are frontline stakeholders in identifying, documenting, and reporting ADRs; however, multiple barriers, such as insufficient training, uncertainty about procedures, and competing clinical demands, contribute to underreporting [3]. Evidence from India mirrors global concerns. Although the Pharmacovigilance Programme of India (PvPI) has expanded awareness and reporting pathways (including the annual National Pharmacovigilance Week), gaps in knowledge and practice remain among healthcare workers [4,5].

Because today’s students are tomorrow’s prescribers, nurses, and pharmacists, strengthening competencies early is a practical route to a sustained reporting culture. Sensitization during undergraduate training is therefore essential to instill early awareness, bridge knowledge gaps, and encourage proactive participation in ADR reporting. In this context, structured sensitization sessions can provide the procedural clarity and motivation needed to improve reporting behaviors during formative years.

Accordingly, this study was designed to (i) assess and compare baseline knowledge and attitudes toward pharmacovigilance and ADR reporting among medical (including Ayurveda) and paramedical (nursing, pharmacy, and allied health sciences) students and (ii) evaluate the impact of a structured pharmacovigilance sensitization session delivered during the fourth National Pharmacovigilance Week (2024) at a tertiary care teaching hospital’s ADR Monitoring Centre in India. We employed a pre-post interventional design to capture change attributable to the educational exposure in a real-world academic setting.

## Materials and methods

This was an interventional study comparing the impact of a pharmacovigilance sensitization session on knowledge and attitude before and after the session amongst medical (including Ayurveda) students and paramedical (nursing, pharmacy, and allied health sciences) students. The study was conducted between September 17 and 23, 2024, at the Department of Pharmacology and ADR Monitoring Centre of DY Patil Medical College and Hospital, Navi Mumbai, Maharashtra, India. The study was approved by the Institutional Ethics Committee for Biomedical and Health Research, DY Patil University School of Medicine (IEC Ref. No: DYP/IECBH/2024/444). All participants provided informed consent before inclusion in the study. Participation was voluntary, and confidentiality of responses was ensured throughout the research process.

Study population

A total of 425 eligible participants were screened from medical (Bachelor of Medicine, Bachelor of Surgery (MBBS), Bachelor of Ayurvedic Medicine and Surgery (BAMS)), paramedical (nursing, pharmacy, physiotherapy, and allied health sciences) undergraduate students currently enrolled at the university.

Eligibility criteria

Inclusion Criteria

Medical or paramedical students who attended both pre- and post-sensitization sessions conducted during the study period, were willing to provide informed consent, and completed both the pre- and post-session questionnaires were included in the study.

Exclusion Criteria

Students with incomplete responses or those who did not meet the inclusion criteria were excluded from the final analysis.

Intervention: sensitization sessions

The intervention consisted of structured educational sensitization sessions focusing on pharmacovigilance and ADR reporting. These sessions were conducted by trained faculty from the Department of Pharmacology and covered topics including the definitions of ADRs, factors contributing to ADRs, national and global ADR reporting systems, the importance of ADR reporting, and the roles of healthcare professionals in pharmacovigilance. Each session lasted approximately 60 minutes and included interactive discussions, case examples, and real-life reporting scenarios.

Data collection tool

The study utilized a pre-validated structured questionnaire designed (validation was done by subject experts in the pharmacovigilance field, including academics and industry personnel) to assess both knowledge and attitude toward pharmacovigilance and ADR reporting (see Appendices). The questionnaire comprised demographic details and two sections: (i) Knowledge section, which consisted of binary (yes/no) and multiple-choice questions with a single correct answer to assess factual knowledge, and (ii) Attitude section, which used a yes/no/don’t know format to evaluate participants' perceptions, beliefs, and willingness regarding ADR reporting practices.

The questionnaire was distributed electronically using Google Forms (Google LLC, Mountain View, California, United States). The link was shared with participants before the intervention (pre-session) and immediately after the intervention (post-session). Responses were securely collected and stored for analysis.

Outcome measures

The primary outcome included assessment of baseline knowledge and attitude toward pharmacovigilance and ADR reporting among medical and paramedical students. The secondary outcome included the evaluation of the impact of the sensitization session on knowledge, measured by comparing pre- and post-intervention correct response rates.

Data management and statistical analysis

All collected data were exported from Google Forms into Microsoft Excel (Microsoft Corporation, Redmond, Washington, United States) for analysis. IBM SPSS Statistics for macOS, Version 29.0 (IBM Corp., Armonk, New York, United States) [6] was used for statistical analysis. Descriptive statistics (frequencies, percentages) were used to summarize demographic data and questionnaire responses. The Chi-square (χ²) test was applied to compare categorical variables between medical and paramedical groups and to assess the statistical significance of pre- and post-intervention changes. Only fully completed questionnaires were included in the final analysis.

Data Coding and Quantification

Knowledge items were designed with one objectively correct response (binary or single-best multiple-choice). Each response was dichotomized as correct = 1 and incorrect or “don’t know” = 0. For items such as the meaning of “pharmacovigilance” or who can report an ADR, the correct option was pre-specified in the answer key based on the Pharmacovigilance Programme of India/World Health Organization definitions. We then summarized counts (n) and percentages (%) of correct responses by group. Post-session “improvement” reflects the increase in the proportion of correct responses from pre-test to post-test within each group; between-group comparisons at each time point used the chi-square test. A p-value of <0.05 was considered statistically significant.

## Results

**Figure 1:**
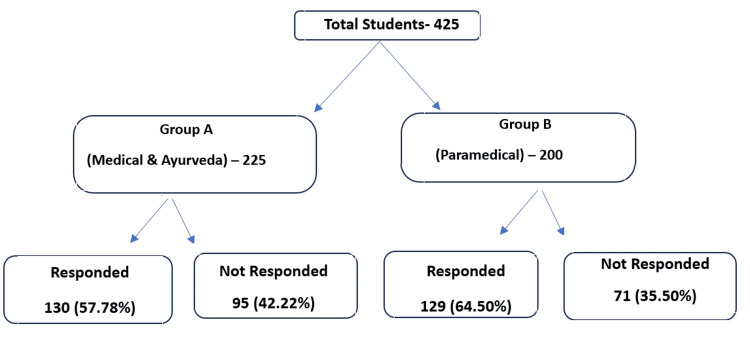
Selection of study participants

Group A consisted of 225 students from the Medical and Ayurveda schools, while Group B included 200 paramedical students from the Nursing, Pharmacy, and Allied Health Sciences schools. The groups include almost equal numbers of participants (Figure 1).

Overall, pretest knowledge related to ADR terminology, ADR reporting, and factors responsible for ADR was better in the medical group as compared to the paramedical group. The difference was statistically significant in knowledge of ADR reporting and ADR forms (82.5% vs 62.8% and 62% vs 48.8%) (Table 1). Amongst all the questions pertaining to knowledge, the minimum correct responses were observed in questions related to the meaning of pharmacovigilance and availability of different ADR forms in both the groups (38% vs 33.3% and 62% vs 48.8%).

**Table 1:** Correct responses to knowledge-based questions in Medical and Paramedical groups (pretest). Note: χ² = Chi-square test; p < 0.05 considered statistically significant. Values are number (percentage) of correct responses per item. Knowledge items were coded as correct = 1, incorrect/‘don’t know’ = 0. Between-group differences were tested using the chi-square test; p<0.05 was considered statistically significant. ADR: Adverse drug reaction; ADE: adverse drug event

SN	Question	Medical Participants (N=130), n (%)	Paramedical Participants (N=129), n (%)	χ² value	p-value
1	Term ADR	101 (77.8%)	94 (72.9%)	0.571	0.4497
2	ADE vs ADR	102 (78%)	94 (72.9%)	0.818	0.3659
3	Factors responsible for ADR	101 (77.8%)	94 (72.9%)	0.571	0.4497
4	Who can report on ADR?	107 (82.5%)	81 (62.8%)	11.433	0.0007
5	Separate ADR reporting forms	80 (62%)	63 (48.8%)	3.726	0.0536
6	Purpose of ADR reporting	95 (73%)	92 (71.3%)	0.075	0.7843
7	Meaning of “Pharmacovigilance”	49 (38%)	43 (33.3%)	0.442	0.5058
8	Agency for drug safety	109 (84.1%)	101 (78.3%)	1.406	0.2359

Table 2 shows the attitude of medical and paramedical groups towards pharmacovigilance (ADR reporting). Regarding the attitude towards ADR prevention, the majority of the medical group (67%) agreed on its preventability, while the majority of the paramedical group (41.1%) were not aware of it. Regarding the role of patient education in ADR prevention, both medical and paramedical participants acknowledged its importance (88.5% and 75.2%, respectively). With respect to the level of social awareness for ADR reporting, both the groups (92.3% and 83.7%) opined that there is insufficient awareness amongst society. The majority of the participants from the medical group (62.3%) agreed that ADR from herbal products should be reported, while 69% from the paramedical group were not in support of it. In the medical group, 90% agreed that the history of ADRs should be taken before starting a medication, while in the paramedical group, there was varied opinion on the same; 40.3% acknowledged its importance, while 33.3% disagreed. The difference between the groups related to attitude was statistically significant.

**Table 2:** Assessment of attitude in Medical and Paramedical groups (pre-test) Note: 3×2 Chi-square test used; values in bold are statistically significant (p < 0.05). ADR: adverse drug reaction; ADE: adverse drug event

SN	Question	Response	Medical (N=130), n (%)	Paramedical (N=129), n (%)	χ² value	p-value
1	Can ADRs be prevented?	Yes	87 (67%)	48 (37.2%)	27.671	<0.00001
		No	20 (15.4%)	28 (21.75%)		
		Don't know	23 (17.7%)	53 (41.1%)		
2	Does patient education help prevent ADRs?	Yes	115 (88.5%)	97 (75.2%)	7.604	0.02227
		No	7 (5.4%)	10 (7.7%)		
		Don't know	8 (6.2%)	22 (17.1%)		
3	Is there general awareness about ADRs?	Yes	9 (6.9%)	12 (9.3%)	7.849	0.01971
		No	120 (92.3%)	108 (83.7%)		
		Don't know	1 (0.77%)	9 (7%)		
4	Should ADRs caused by herbal products be reported?	Yes	81 (62.3%)	4 (3.1%)	107.810	<0.00001
		No	7 (5.4%)	89 (69%)		
		Don't know	42 (32.3%)	36 (27.9%)		
5	Should HCPs ask about ADR history before prescribing?	Yes	117 (90%)	52 (40.3%)	62.530	<0.00001
		No	3 (2.3%)	43 (33.3%)		
		Don't know	10 (7.7%)	34 (26.4%)		

As shown in Table 3, improvement was seen in all the sections of the questionnaire (ADR terminologies, factors, reporting, and Pharmacovigilance) in both groups. The difference between the two groups was highly significant (p <0.001) in questions related to the availability of different ADR forms (98% and 78.8%) and the meaning of the term “pharmacovigilance” (87.7% and 66.3%). The maximum improvement was seen in questions related to ADR reporting in medical and paramedical groups (95.6% and 81.3%), and in ADR terminologies (86.2% and 74.3%). Lesser improvement was observed in questions about the purpose of ADR reporting (42.9% and 24.3%) and the global agency associated with drug safety and factors affecting ADR (52.4% and 39.3%).

**Table 3:** Shift of incorrect to correct responses following sensitization session - impact Note: Chi-square test used; p < 0.05 considered significant; values in bold denote statistical significance ADR: adverse drug reaction; ADE: adverse drug event

SN	Question	Medical (N=130), n (%)	Paramedical (N=129), n (%)	χ² value	p-value
1	Term ADR	25 (86.2%)	26 (74.3%)	1.406	0.2359
2	ADE vs ADR	19 (67.9%)	26 (74.3%)	0.442	0.5058
3	Factors responsible for ADR	14 (48.3%)	18 (51.4%)	0.108	0.7423
4	Who can report on ADR?	22 (95.6%)	39 (81.3%)	3.803	0.0511
5	Separate ADR forms	49 (98%)	52 (78.8%)	8.204	0.0042
6	Purpose of ADR reporting	15 (42.9%)	9 (24.3%)	3.105	0.0779
7	Meaning of “Pharmacovigilance”	71 (87.7%)	57 (66.3%)	9.083	0.0026
8	Global agency for drug safety	11 (52.4%)	11 (39.3%)	1.210	0.2711

## Discussion

Reporting ADRs is a critical aspect of the pharmacovigilance program. The spontaneous reporting system plays a significant role in documenting ADR and identifying new reactions associated with the drugs. Pharmacovigilance and ADR reporting are important competencies all healthcare school students need to obtain before they graduate and become involved in clinical practice as healthcare professionals [7]. Therefore, educating healthcare students in the schools of medicine, pharmacy, dentistry, or nursing and involving them early in clinical practice to prescribe, administer, and/or monitor medications are essential to ensure the safe use of medications [8].

In the present study, we aimed to assess the understanding of medical and paramedical students on ADR reporting, factors responsible for ADR, social awareness, and their attitude towards the same in a tertiary care hospital. This study shows that medical students presented a higher level of knowledge in answering questions related to ADR terminologies, ADR reporting, factors responsible, purpose of ADR reporting, and pharmacovigilance. The difference was statistically significant compared to the paramedical group. This is in line with a study conducted by Rehan et al. [9].

This could be because of inadequate or no training on this topic, and being inadequately prepared during their education for their role in monitoring and reporting ADRs [10,11]. Previous studies have also shown that pharmacy students have insufficient knowledge of pharmacovigilance and ADR reporting. For example, a systematic review conducted in 2020 to evaluate the knowledge, attitude, and perception of pharmacovigilance and ADR reporting in healthcare students and identify the current need for education/training on pharmacovigilance and the research need to improve it [12].

In this study, attitude was measured by using multiple-choice questions (yes/no/don’t know). It was observed that medical students had a more positive attitude towards all aspects of PV and ADRs, while in the paramedical group, the majority had chosen the "don’t know" option, which could be because of a lack of knowledge or interest. This is in line with a similar study conducted amongst healthcare students in a tertiary care teaching hospital, Telangana, India [13]. However, the studies conducted in 2020 by Alwhaibi et al. [12] and in 2018 by Singh et al. [14] found that paramedical (Pharmacy & Nursing) students were better trained in pharmacovigilance and ADR reporting.

In this study, we have observed an overall good impact on the knowledge of pharmacovigilance in both groups. Less improvement was seen in factors responsible for ADR, the global agency for ADR reporting, and the purpose of ADR reporting. This indicates that there is a need for regular sensitization of paramedical students and to encourage them in ADR reporting. This is in accordance with a study conducted in 2019 that assessed knowledge gained at the end of a pharmacovigilance sensitization workshop [15].

A 2020 Saudi study found pharmacy students had stronger knowledge, attitudes, and perceptions of pharmacovigilance than peers from other health colleges, underscoring the need to integrate pharmacovigilance training into curricula [12].

The strength of our study lies in the comprehensive assessment of knowledge and attitude regarding pharmacovigilance (ADR reporting) among future healthcare providers. The questionnaire comprehensively covered a wide range of pharmacovigilance (ADR reporting) knowledge topics to ensure a thorough grasp of the problem. This study assessed the knowledge and attitude of medical and paramedical students, providing valuable insights into the variations and facilitating the precise adaptation of more effective educational interventions.

A significant limitation is its smaller sample size, focusing only on students and not involving healthcare staff who are actual healthcare providers. Practices could not be assessed for the same reason. As this was a single-center study, the sample cannot accurately represent every medical and paramedical student in different institutions or regions, which might limit the extent to which the results can be applied. However, despite these limitations, the findings can guide the creation of focused educational initiatives aimed at enhancing pharmacovigilance practices and improving patient care in general.

## Conclusions

This questionnaire-based study demonstrates that medical students possessed comparatively higher baseline knowledge of pharmacovigilance and ADR reporting than paramedical students. Attitude analysis revealed that medical students displayed a more positive outlook toward ADR prevention, patient education, and the importance of reporting, while paramedical students frequently expressed uncertainty. Following a structured sensitization session, both groups exhibited significant improvement in knowledge, particularly in core domains such as ADR reporting, the availability of reporting forms, and the meaning of pharmacovigilance. Notably, a significant percentage of initially incorrect responders shifted to correct responses after the intervention, highlighting the value of targeted teaching in bridging knowledge gaps.

Overall, the findings underscore the importance of regular pharmacovigilance sensitization activities for healthcare students. Such interventions not only enhance knowledge but also positively influence attitudes, thereby strengthening the foundation for robust ADR reporting practices. Integrating structured pharmacovigilance training into the curricula of both medical and paramedical streams is recommended to ensure the preparedness of future healthcare professionals in promoting drug safety and minimizing ADR-related risks.

## Appendices

Questionnaire

Section A. Knowledge-Based Questions

Q1. What does “ADR” stand for?

a) Adverse Drug Reaction

b) Adverse Drug Response

c) Adverse Dosage Result

d) Don’t know

Q2. Are Adverse Drug Event (ADE) and Adverse Drug Reaction (ADR) same?

a) Yes

b) No

Q3. Which of the following factors commonly contribute to ADRs?

a) Polypharmacy

b) Drug interactions

c) Genetic variability

d) All of the above

Q4. Who can report an ADR in India?

a) Only doctors

b) Only patients

c) All healthcare personnels

d) All healthcare personnels and patients

Q5. Are there separate ADR reporting forms available for healthcare professionals and patients in India?

a) Yes

b) No

c) Don’t know

Q6. What is the primary purpose of ADR reporting?

a) To penalize prescribers

b) To improve patient safety and drug monitoring

c) To identify counterfeit drugs

d) Don’t know

Q7. What does “Pharmacovigilance” mean?

a) Vigilance about science of pharmacology

b) Detection, assessment, and prevention of adverse drug effects

c) Ensuring drug affordability

d) Don’t know

Q8. Which international agency is primarily responsible for global drug safety monitoring?

a) US-FDA

b) WHO

c) EMA

d) Don’t know

Section B. Attitude-Based Questions

Q1. Can ADRs be prevented?

Yes / No / Don’t know

Q2. Does patient education help prevent ADRs?

Yes / No / Don’t know

Q3. Is there general awareness about ADRs?

Yes / No / Don’t know

Q4. Should ADRs caused by herbal products be reported?

Yes / No / Don’t know

Q5. Should HCPs ask about ADR history before prescribing?

Yes / No / Don’t know
